# Tremella Polysaccharide Has Potential to Retard Wheat Starch Gel System Retrogradation and Mechanism Research

**DOI:** 10.3390/foods12163115

**Published:** 2023-08-18

**Authors:** Jiaxun Wang, Shanshan Zhang, Nan Wang, Hongxiu Fan, Hanmiao Wang, Tingting Liu

**Affiliations:** 1 College of Food Science and Engineering, Jilin Agricultural University, Changchun 130118, China; 13154368138@163.com (J.W.); winkyshanshan@163.com (S.Z.); 18943920137@163.com (N.W.); fanhongxiu20071105@126.com (H.F.); whm15567140581@163.com (H.W.); 2 Scientific Research Base of Edible Mushroom Processing Technology, Integration of Ministry of Agriculture and Rural Affairs, Changchun 130118, China

**Keywords:** wheat starch, tremella polysaccharide, retrogradation properties

## Abstract

This study investigated the effects of adding different concentrations of TP (tremella polysaccharide) on the water distribution, rheological, thermal, microstructure, and retrogradation properties of WS (wheat starch) gels. The results showed that the starch aging increased during storage, and the addition of TP reduced the rate of change of the elastic modulus of the starch gel and delayed the short-term aging of WS. In the same storage period, the hardness value of the gel decreased and the texture became softer with the increase in the mass fraction of TP. TP increased the T_0_ (starting temperature) of the system and decreased the enthalpy of retrogradation (ΔH_r_). No new groups were formed after the retrogradation of the compound system, the hydrogen bonding force increased with the increase in polysaccharide, and the relative crystallinity and the degree of ordering of the system decreased. The addition of TP increased the content of bound water and immobile water, decreased the content of free water, and increased the gel water-holding capacity, indicating that it could effectively inhibit the long-term retrogradation of WS. The findings provide new theoretical insights for the production of starch-based foods.

## 1. Introduction

Foods produced with wheat flour as the main raw material, such as bread, Chinese steamed breads, and cake, are the favorite staple foods of people all over the world. However, the aging, hard texture, and poor taste of wheat staple food during storage significantly affect the product quality. The reason for this phenomenon is the aging of the wheat starch, which is the component with the highest content in wheat flour and accounts for 85% of the dry weight [1]. Starch retrogradation is the reverse process of pasting, where dissolved and swollen starch molecules are rearranged and combined, and the disordered structure is rearranged into an ordered double-helix structure to form a precipitate or gel [2]. Starch retrogradation can cause quality changes in foods in storage, such as hardening of buns, noodles, and cake. However, moderate regrowth can improve the textural and sensory properties of some starchy foods. For example, in the case of rice noodles and vermicelli, moderate retrogradation affects their textural properties and gives them better elasticity and chewiness [3]. Therefore, it is important to investigate the aging mechanism of starch to study retrogradation of rice and flour products and the actual production of food.

Starch is widely used in the food industry as a gelling agent, stabilizer, thickener, and water retention agent [4]. However, natural wheat starch suffers from poor solubility, easy aging after pasting, and dehydration and shrinkage, which delays its development in the food and other industries [5,6]. At present, the method of physical modification of starch is not only friendly to the environment, but also easy to implement. In particular, the effects of non-starch polysaccharide on the gel properties and retrogradation characteristics of starch have attracted much attention. It was found that the interaction between non-starch polysaccharides and starch had an effect on their aging properties, rheological properties, and pasting properties. Using rheometry, a food texture analyzer, low-field nuclear magnetic resonance (LF-NMR), and X-ray diffraction (XRD), Zhou et al. [7] demonstrated that the addition of Laminaria japonica polysaccharides improved the microstructure of WS gels, reduced the hardness of the starch gels during storage, and effectively inhibited starch retrogradation. This is due to the interaction between LJP and amylose, which reduces the amount of extracted amylose, and secondly, a large number of hydroxyl groups have a certain inhibitory effect on the hydrogen bonding between starch chains. Sodium alginate [8] and galactomannan [9] both inhibited straight-chain starch leaching or co-crystallization between straight-chain and branched-chain starch to retard starch retrogradation. Several authors have shown that xanthan gum inhibits both short- and long-term retrogradation of rice starch and corn starch due to the fact that the non-starch polysaccharides in the mixed system compete with starch molecules for water absorption, limiting water flow and thus affecting the water-holding capacity of the gel during storage [10,11].

Different hydrocolloids may also have different effects on starch due to differences in their origin and structure. The TP selected for this experiment was a heteropolysaccharide obtained by isolation and purification from the seeds of the tremella fruiting body, which is a combination of ten monosaccharides including glucose, galactose, arabinose, rhamnose, and ribose, with mannose as the main chain [12]. TP is a natural edible fungus with medicinal and edible origins, and is highly loved by the Chinese people due to its nutritional value and flavor. Studies have reported that TP has good biological activities such as antioxidant, skin protection, immunomodulatory, anti-tumor, and hypoglycemic effects [13,14,15,16]. Because of its wide availability, natural status, and high degree of safety, it is often used in the food industry as a thickening agent and stabilizer. Other hydrocolloids such as sodium alginate (SA), which is a natural linear polysaccharide found in brown algae such as kelp and gulfweed, are connected by the (1 → 4) bond of β-D-mannuronic, M and α-L-guluronic, G [8]. However, the content of SA added to food is limited because long-term consumption will increase the burden on the gastrointestinal tract and affect the absorption of nutrients. For TP, there is no limit on the amount used in the food industry. Xanthan gum (XG) is the largest extracellular polysaccharide produced in the world. It is composed of D-glucose group on the main chain of cellulose and three sugar units on the side chain [10]. It is an anionic acid polysaccharide. However, the human body cannot basically digest and absorb XG, and TP has higher nutritional value. Liu et al. [17] showed that TP can enhance the water-holding capacity of gels and significantly affect their dynamic rheological properties. Yang et al. [18] found that TP could inhibit the swelling of potato starch granules and the leaching of straight-chain starch in a compound system. However, there are few studies on the retrogradation properties and interaction mechanism of TP on starch gels. Therefore, in this study, we investigated the effects of different additions of TP on the aging properties of WS by rheological analysis, DSC, XRD, and other experimental methods, and analyzed the mechanism using the LF-NMR method to provide a theoretical basis for further enrichment of non-starch polysaccharides for starch quality improvement.

## 2. Materials and Methods

### 2.1. Materials and Reagents

Wheat starch (purity 99.99%) was purchased from Yuanye Biotechnology Co., Ltd., Shanghai, China. (straight-chain starch content, moisture content, lipid content, protein content, and ash content were 24.28%, 13.02%, 0.49%, 0.71%, and 0.41%, respectively).

TP: Tremella was purchased from Wal-Mart Supermarket, Xunche Plaza Store, Changchun, China, and TP was made by the laboratory.

### 2.2. Instruments and Equipment

TA.XT Plus texture analyzer, Beijing Weixun Ultra Technology Instrument Technology Co., Ltd., Beijing, China; DHR-1 rheometer Waters Group, Milford, MA, USA; Discovery DSC 25XX differential calorimetry scanner, TA, New Castle, DE, USA; MesoMR23-040V-I low-field NMR instrument Shanghai Newmark Electronic Technology Co., Ltd., Shanghai, China; Phenom Pro scanning electron microscope; IR Prestige-21 Fourier Transform Infrared Spectrometer, Shimadzu, Kyoto, Japan; MiniFlx 600 Benchtop X-ray Diffractometer.

### 2.3. Sample Preparation

WS (6%, *w*/*v*)-TP (0, 0.5, 1, 2, 3%, *w*/*w*, percentages based on WS) gels were prepared as follows: TP was dissolved in deionized water to which WS was added to obtain a homogeneous solution, which was stirred at 25 °C for 30 min to obtain a mixture. The gel was then formed by stirring in a water bath at 95 °C for 30 min and then cooled to room temperature. All the gels were placed in a uniform container, and the gel was made into a cylinder with a diameter of six centimeters and a height of eight centimeters; the gel was stored in a 4 °C refrigerator for 1, 7, and 14 days. A portion of the gel was removed and lyophilized and ground through a 100-mesh sieve.

### 2.4. Determination of Gel Texture

The fresh starch paste prepared in Section 2.3 was refrigerated at 4 °C for 1, 7, and 14 d. The starch gels were tested for hardness using a food texture analyzer. Measurement conditions: P/0.5 probe, pre-test rate of 1.5 mm/s, test rate of 2.0 mm/s, post-test rate of 2.0 mm/s, test distance of 10.0 mm, and trigger force of 5 g.

### 2.5. Dynamic Time Scan Determination

The starch paste prepared in Section 2.3 was cooled to room temperature and placed on a rheometer with a 40 mm diameter plate jig and a gap of 1000 μm to start the dynamic time scan experiment by referring to the methods of Zhang Dandan et al. [19]. The test temperature was 4 °C, frequency 1 Hz, scanning strain 1%, and the change in the elastic modulus G’ of the sample was measured over 2 h.

### 2.6. Determination of Thermodynamic Properties

According to Zhang et al. [20], 0.5 g of wheat starch of a certain weight and TP of 0%, 0.5%, 1%, 2%, and 3% of its dry base weight were separately dispersed in 1 mL distilled water, and 10 mg was weighed and put into the crucible after being evenly mixed. The gelatinization enthalpy (ΔH_g_) was determined by the first programmed temperature measurement of the crucible after 24 h of equilibrium at room temperature. The set procedure was to increase temperature from 20 °C to 100 °C at a rate of 10 °C/min. After the gelatinized samples were cooled and stored at 4 °C for 1, 7, and 14 days, they were taken out successively for a second thermal scan to determine the enthalpy of retrogradation (ΔH_r_) of the samples. The aging rate was the ratio of enthalpy of retrogradation to enthalpy of gelatinization (ΔH_r_/ΔH_g_).

### 2.7. Fourier Infrared Spectrum Scanning

The starch gel was refrigerated at 4 °C for 1, 7, and 14 days, and then freeze-dried and ground into powder, which was then put through a 100-mesh sieve for use. Samples of 1 mg and 50 mg potassium bromide were weighed, ground, and mixed evenly, and then vacuumed at 15 MPa. Excluding the background of potassium bromide flakes, the determination range was 4000~400 cm^−1^; 64 scans were performed with a resolution of 4 cm^−1^, and Origin 2021 was used for data processing to obtain infrared spectra. OMNIC 8.0 was used to deconvolute the infrared spectrum in the range of 1200 cm^−1^ to 900 cm^−1^, and the enhancement factor was set to 1.9 and the half-peak width to 40 cm^−1^.

### 2.8. X-ray Diffraction Measurement

The ground sample in Section 2.3 and ungelatinized raw wheat starch were used for XRD detection. The experimental parameters of the diffractometer were set as follows: voltage was 40 kv and current was 40 mA. The scanning range of diffraction angle 2θ was 5°~40°, the scanning rate was 4°/min, and the scanning step length was 0.02°. MDI jade 6.0 software was used to calculate the relative crystallinity.

### 2.9. Measurement of Water Migration Change

Measurements were taken using slight modifications to the method of Wang et al. [21]. The gel samples stored for different days were placed in glass NMR tubes, and the transverse relaxation time T2 of the samples was measured by CPMG (multi-pulse echo sequence). The main parameters were set as: Rf signal frequency offset O_1_ = 376,786.36 Hz, sampling number TD = 1,000,054, P_1_ = 3.00 μs, P_2_ = 6.00 μs, SW =200 kHz, TE = 0.5 ms, T_w_ = 7500 ms, RFD = 0.08 ms, RG1 = 20.0 db, DRG1 = 3, cumulative times NS = 4, echo number NECH = 10,000. The signal was collected by NMRAS120 analysis software, and the data were normalized after inversion.

### 2.10. SEM

The fresh starch paste prepared in Section 2.3 was refrigerated at 4 °C for 1, 7, and 14 d. The gel was fixed on the sample cup and the sample was pre-cooled at −20 °C. After freezing, the sample was placed in a sample tank, so that the internal environment of the cabin door was a vacuum ring. The images were observed and photographed under an electron cryopreservation microscope. The test conditions were voltage value: 5 kV; magnification: 44.8 μm, 6000 times.

### 2.11. Statistical Analysis

SPSS Statistics 23.0 software was used to process the data, and the significant differences were determined using ANOVA. *p* < 0.05 was considered significant. Origin 2021 was used for software mapping.

## 3. Results and Discussion

### 3.1. Gel Strength Analysis of the Compound System of WS and TP

**Figure 1:**
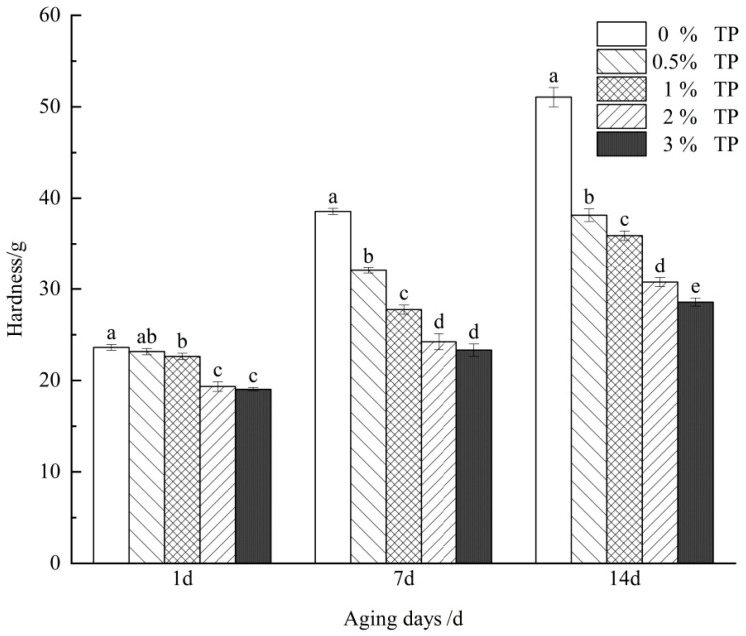
Parameters of hardness of retrogradation wheat starch/different concentrations of TP mixed systems. Different letters on the same day indicate significant differences (*p* < 0.05).

The aging degree of starch gel during storage is closely linked to its hardness, which can be characterized by the gel hardness value in the food physical property instrument [22]. As shown in Figure 1, when all samples are stored at 4 °C, the gel hardness increases with the extension of storage days, which is consistent with the study of Chen et al. [23]. The aging process of starch causes the increase in starch gel hardness, which is related to the recrystallization of starch [24]. Compared with the pure starch, the hardness of the samples supplemented with TP decreased, and the hardness was negatively correlated with the content of TP. On the one hand, it may be that polysaccharide obstructs amylose aggregation and rearrangement, reduces the force between amylose, slows down the aging degree of gel, and makes the gel softer [25]; on the other hand, it may be that polysaccharide replaces part of starch, which weakens the total amount of starch and reduces the strength of gel.

### 3.2. Dynamic Time Scan Analysis

Gelatinized starch undergoes aging during storage, which can be generally divided into short-term aging and long-term aging [26]. Short-term aging occurs in the initial stage after starch gelatinization, which is generally completed within a few hours or ten hours. The gel network structure is formed mainly through hydrogen bond aggregation among amylose molecules, which enhances the elasticity of starch, and is manifested as an increase in energy storage modulus in dynamic time scanning tests [27].

**Figure 2:**
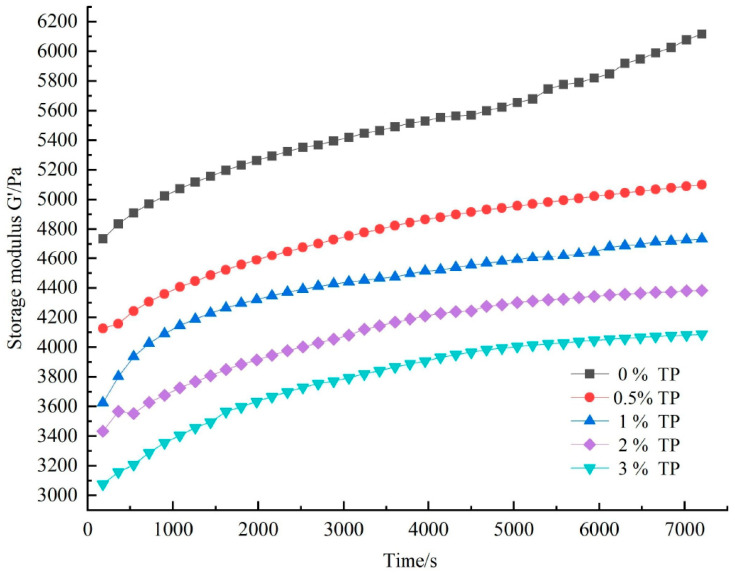
Elasticity modulus as a function of time for WS/different concentrations of TP mixed systems.

Figure 2 shows the time-varying curve of the energy storage modulus G’ of the tremella polysaccharide–wheat starch compound system with different concentrations after aging for 2 h at 4 °C. At the initial stage of the experiment, G’ of all gel samples increased rapidly, and the compound system with TP increased slowly with aging time. This may be due to the combination of amylose and TP during gelatinization, which slowed down the aggregation and rearrangement rate. Similar results have been seen in the Laminaria japonica polysaccharide–wheat starch gel system [7]. Alternatively, hydrophilic TP binds with water to restrict water flow in the system, thus affecting the amylose rearrangement rate [28]. With the increase in TP content, the G’ value decreased successively, and the change rate showed a decreasing trend, which indicated that adding TP can inhibit the aging behavior of WS. In addition, the effect was more obvious with the increase in the amount added.

### 3.3. Thermodynamic Properties of Wheat Starch–Tremella Polysaccharide Compound System

**Figure 3:**
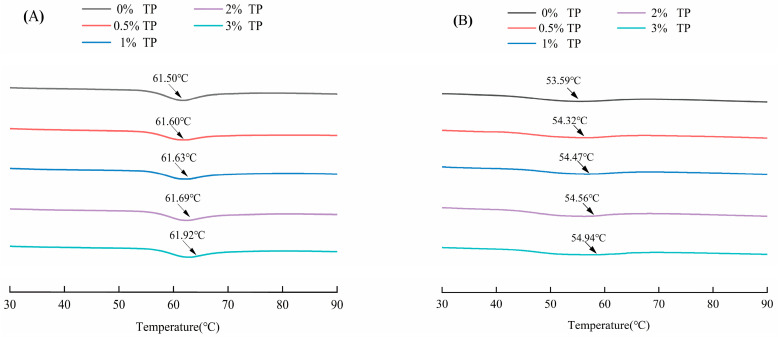
Effect of TP on DSC curves of fresh (0 d) and stored (14 d) WS gel samples: (**A**) gelatinization curve of the gel system, and (**B**) retrogradation curve on the 14th day of storage.

The enthalpy value (∆H_r_) represents the energy required to melt the crystalline structure, and the larger the enthalpy value, the higher the degree of starch recrystallization and the greater the degree of aging [10]. The thermodynamic analysis of each sample during storage is shown in Table 1. No obvious enthalpy of aging was found in the samples stored for 1 d, but the aging degree of samples stored for 7 d and 14 d was found to increase with the increase in enthalpy value. This also represented the formation of an ordered and dense structure between WS molecules through hydrogen bonding, leading to the need for more energy to destroy the double-helix structure. The peak temperature T_p_ of starch after storage aging is about 50 °C, which is associated with the dissociation peak of amylopectin aging [29]. Figure 3 shows the DSC curve of the system for 14 days of gelling and refrigeration. After storage, the initial temperature T_0_ and peak temperature T_p_ show a decreasing trend, but the enthalpy value ∆H increases gradually, indicating that the degree of starch aging increases and the energy of melting amylopectin crystallization increases.

The ΔH_g_ of wheat starch decreased obviously with the increase in the supplemental level of TP. A similar effect was found [30] in the potato starch–tremella polysaccharide composite system. However, the addition of xanthan gum can significantly increase ΔH of cassava starch, while carrageenan has no significant effect on ΔH of cassava starch. This may be related to the type of hydrophilic colloid. ΔH_r_ increases gradually with the extension of refrigeration time, indicating that starch aging degree becomes greater during refrigeration. Table 1 shows that the addition of TP decreased ΔH_r_ and retrogradation percentage of wheat starch, and the greater the TP added, the more ΔH_r_ and retrogradation percentage decreased, and the more obvious the degree of aging delay. This is because TP has good water-holding capacity, which increases the concentration around starch molecules, hindering the migration of molecular chains and delaying the recrystallization of starch. In addition, TP has a certain embedding effect on starch particles, and TP interacts with leaking amylose through hydrogen bonding, interfering with the formation of amylose crystal nuclei and thus inhibiting the aging of starch [22,31]. The retrogradation percentage decreased from (55.00 ± 0.08)% to (37.44 ± 0.06)% when the proportion of TP increased from 0% to 3% and the storage lasted for 7 days. When the storage was 14 days, the retrogradation percentage decreased from (68.87 ± 0.10)% to (50.25 ± 0.41)%. The experiment result shows that TP can inhibit the aging of WS to a certain extent.

### 3.4. Fourier Infrared Spectrum Scanning Analysis of Mixed Systems

**Figure 4:**
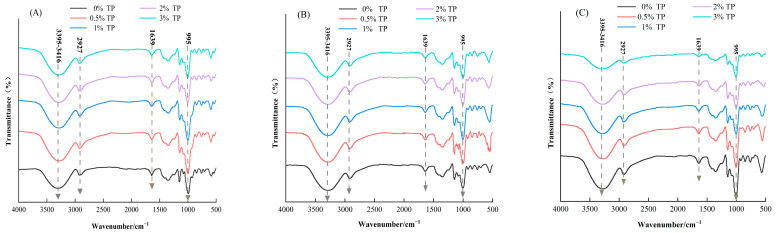
Effects of TP on structure of WS gels: (**A**–**C**) correspond to the FTIR spectrum of 1 d, 7 d, and 14 d samples, respectively.

In this research, the infrared spectrum was used to reflect the short-range orderliness of WS structures, and the results are shown in Figure 4. The absorption peak of the WS-TP compound system appears near the range of 3395 to 3416 cm^−1^, which is a typical stretching vibration peak of hydroxyl groups. As both WS and TP belong to polysaccharide macromolecules and contain a large quantity of hydroxyl groups, a large absorption peak appears here. The absorption peak near 2928 cm^−1^ is related to the asymmetric tensile vibration of -CH [32]. At 1639 cm^−1^, the peak value of the composite system containing TP decreased, and the effect became more obvious with the increase in TP content. For wheat starch, the peak at 1639 cm^−1^ is caused by the O-H bending vibration of water molecules in the sample [33] and is associated with the water content of the sample. TP is a hydrophilic macromolecule that competes with starch for water in the mixing system, thus reducing the water content of starch gel, and leading to a decrease in the peak value at 1639 cm^−1^. As shown in Figure 4, no new absorption peaks appeared in the map of storage for 1, 7, and 14 days, indicating that WS and TP were interacting with each other through non-covalent interactions such as hydrogen bonding.

The absorption peak intensity near (1054/1021) cm^−1^ and (995/1022) cm^−1^ can reflect the short-range orderliness of the system and the internal variation of the double helicity [34]. As can be seen from Table 2, the ratio of (1054/1021) cm^−1^ and (995/1022) cm^−1^ of WS stored for 1 day was 1.116 and 1.323, respectively. After 14 days of storage, the values of (1054/1021) cm^−1^ and (995/1022) cm^−1^ were significantly increased to 1.214 (*p* < 0.05) and 1.424 (*p* < 0.05). The results showed that the WS aged during storage and the double-helix packing density became more and more compact on the surface of starch grains. The ratio of (1054/1021) cm^−1^ and (1021/993) cm^−1^ of the compound system decreased gradually with the increase in TP mass fraction, indicating that TP can reduce the degree of order of the system and effectively delay the short-term and long-term aging of starch.

### 3.5. X-ray Diffraction Analysis

**Figure 5:**
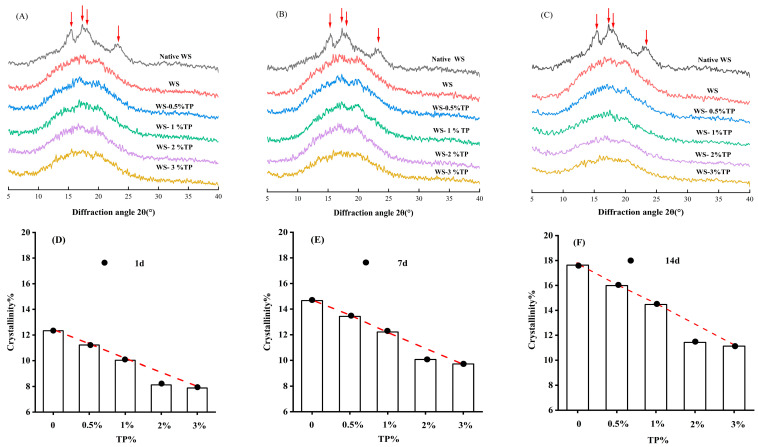
Effects of TP on crystallinity in 1 d, 7 d, and 14 d WS gel samples: (**A**–**C**) correspond to the XRD patterns of 1 d, 7 d, and 14 d samples, respectively; (**D**–**F**) correspond to the crystallinity of WS gel stored for 1 d, 7 d, and 14 d samples, respectively.

XRD can characterize the long-range orderliness of WS. As shown in Figure 5, XRD was devoted to analyzing the effect of different amounts of TP on WS crystallization during aging (1 d, 7 d, 14 d). It can be obviously seen from Figure 5 that raw wheat starch has a strong absorption peak at around 15° and 23°, and a double peak at around 18°, which is a typical A-type structure. After gelatinization and storage, strong absorption peaks appeared in all samples at 17° and 20°, indicating that the crystal structure of starch gels was destroyed during gelatinization, transforming from type A structure to type B structure. The B-type structure is the characteristic diffraction peak of starch after aging, and it is a double-helix structure formed between amylose and amylopectin, which can reflect the degree of starch coagulation [8,35]. In this study, the coagulation phenomenon of WS gel was verified by the increase in peak strength at 2θ = 17° with the extension of storage days. Compared with single wheat starch, no new crystals were produced in the compound system, indicating that the addition of TP had no effect on the crystal type of starch. Compared with pure wheat starch, the diffraction peak intensity near 17° was weaker in the compound system with TP, indicating that TP could inhibit the recrystallization of starch.

The crystallinity of WS stored for 1 d, 7 d, and 14 d is shown in Figure 5. With the extension of storage time, crystallinity increased significantly, which is because starch crystals change from an amorphous state to a polycrystalline state during storage [36]. The crystallinity of starch can reflect the degree of aging of WS. The addition of TP reduces the crystallinity of gel during each storage day, indicating that TP inhibits the transformation of starch crystal from an amorphous state to a polycrystalline state. This may be caused by the interaction between the hydroxyl group and amylose in TP, which to some extent prevents the combination of amylose with lipids or other small molecules, thus curbing the formation of V-shaped crystals. Alternatively, the hydroxyl group in TP interacts with WS and water molecules through hydrogen bonding, hindering the interaction between starch chains and leading to the reduction in the relative crystallinity of starch. Related studies have found that the addition of laminaria polysaccharide or inulin [7,35] can reduce starch crystallinity, indicating that TP has a similar influence on inhibiting amylopectin recrystallization and starch aging.

### 3.6. Determination of Water Migration Change Rule

Starch is often accompanied by water changes in the retrogradation process, and LF-NMR can quickly analyze the water distribution in the system [37]. It was speculated in Section 3.3 and Section 3.4 that high quality fraction TP had higher water-holding capacity than low quality fraction TP. Therefore, high quality fraction TP had a more obvious effect on inhibiting wheat starch gel. Starch is often accompanied by water changes in the retrogradation process, and LF-NMR can quickly analyze the water distribution in the system [37].

In this work, LF-NMR was used to further analyze the changes in water migration in WS gel during the retrogradation process to verify the above speculation. The transverse relaxation time T_2_ of LF-NMR can reflect the water migration of the starch gel system [38], which can characterize the degree of binding between the sample and water. The lower the value of T_2_, the closer and higher the binding between the substrate and water molecules [39]. In the starch gel system, some water molecules are combined with macromolecular groups to form binding water (T_2b_, T_21_), and the relaxation time is within 0.1~10 ms. There was unmovable water (T_22_) between amylose and amylopectin, and the relaxation time was within 10~100 ms. Relaxation time greater than 100 ms is considered to be free water T_23_ [40].

**Figure 6:**
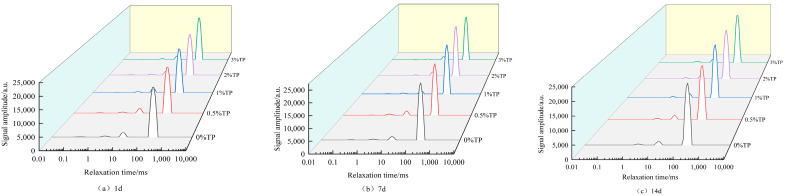
Effect of TP with different mass fractions on water migration of WS gel for different storage days. (**a**), (**b**) and (**c**) are respectively water migration images of WS gel stored for 1, 7 and 14 days.

The water migration changes of the compound system with different mass fractions of TP during the 1th, 7th, and 14th days of storage are shown in Figure 6 and Table 3. Compared with CS, the T_2_ value of the system decreases with the addition of TP, indicating that the system is more closely bound to water molecules. The signal peak at the front end gradually decreases and disappears with the extension of storage time, which means that the close-knit structures in part of the system are degraded. The T_22_ and T_23_ values of all samples decreased with the increase in storage days. This indicates that the binding between starch and water molecules is gradually enhanced, and the leakage of amylose aggregates and rearranges, limiting the mobility of water [39]. As a result, starch gels develop a denser structure during aging, which is compatible with the trend found in the TPA hardness test. According to Table 3, the T_21_ and T_23_ values of gels decreased with the increase in TP mass fraction; thus, system showed that a high TP mass fraction can enhance the water-holding capacity of gels and reduce the mobility of water molecules.

**Figure 7:**
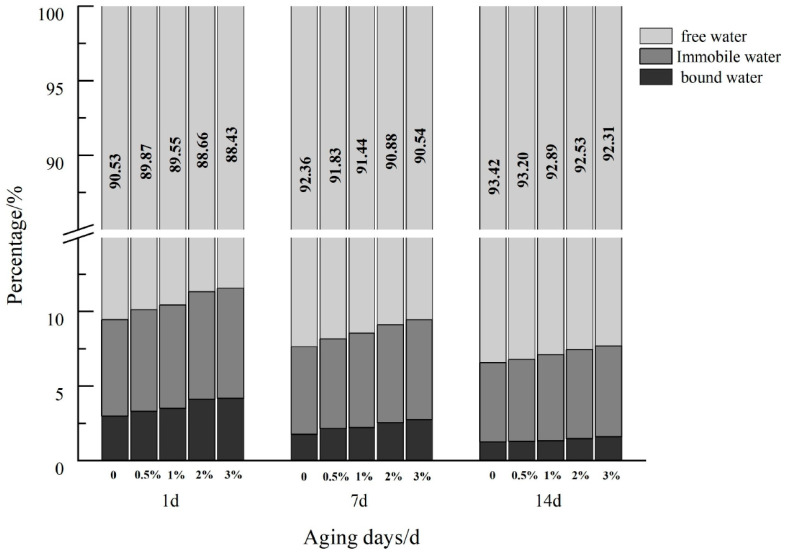
Peak area ratio of retrogradation wheat starch/different concentrations of TP mixed systems.

The variation in the peak area and peak area ratio of each peak can be obtained by integrating the relaxation spectra. The relative content of water molecular mobility is proportional to the corresponding peak area ratio. Figure 7 shows the peak area ratio diagram of the aging process of the mixed system of WS and TP of different concentrations. In the compound system, the content of free water is the highest, followed by unmovable water, and the content of bound water is the lowest. With the increase in retrogradation time, the content of bound water and immovable water decreased gradually, while the content of free water increased gradually. It was proven that, in the storage process, the hydrogen bond between starch chains was strengthened, forcing the hydrogen bond between starch chains and water molecules to weaken, resulting in water precipitation, partial binding of water to free water, and decreased gel water retention [41,42]. Within the same storage time, with the increase in TP concentration, the free water content gradually decreased, which is consistent with the study of Ma et al. [43]. This phenomenon indicated that TP enhanced the water retention of starch gel, reduced the dehydration shrinkage of the system, and effectively slowed the aging process of starch.

### 3.7. SEM

**Figure 8:**
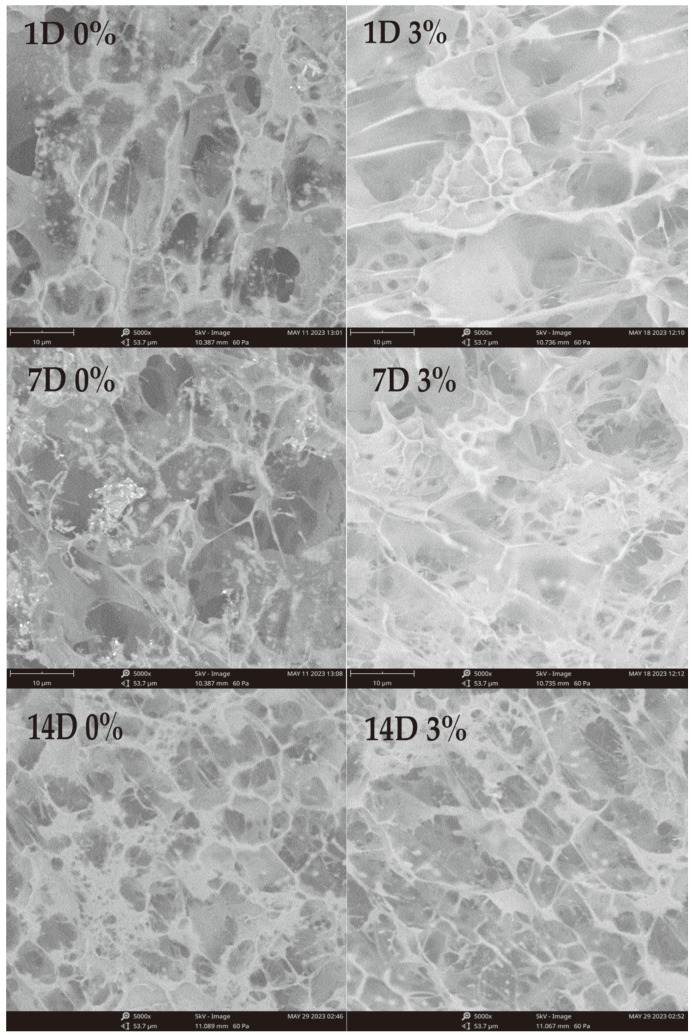
Effect of no TP and addition of 3% TP on the microstructure of WS gel preserved for 1 d, 7 d, and 14 d.

It was observed by SEM that the gelatinized gel aggregated to form a dense three-dimensional network structure during the cooling process. As shown in Figure 8, all samples exhibited a honeycomb-like structure. Along with storage time extension, the aging degree of starch gel deepens to form a denser structure, which can increase the hardness of the gel. The starch gels with TP added have larger pores and more uniform network distribution, and the gel hardness is reduced, which is consistent with the gel strength test results in TPA. In the Mesona Chinensis Benth polysaccharide–potato starch complex system, the phenomenon of enlarged gel pore size was also observed after polysaccharide was added, and Luo et al. [44] proved that polysaccharide can improve the microstructure of gel at an appropriate concentration. TP can obviously improve the microstructure of aging starch gel. This may be because the size of gel pores during gelatinization is related to water distribution [42,43] and TP contains a large number of hydroxyl and hydrophilic groups, which is conducive to the formation of aggregates by hydrogen bonding of amylose, thereby improving the gel structure of WS. On the other hand, hydroxyl groups interact with amylose and amylopectin to strengthen intermolecular hydrogen bonds to form gel matrix structures. It was observed using scanning electron microscopy that the gelatinized gel aggregated to form a dense three-dimensional network structure during the cooling process.

## 4. Conclusions

This paper focuses on the effects of TP concentration on the water migration and aging characteristics of the WS gel system. The results showed that TP has the potential to retard the retrogradation of the wheat starch gel system. The elastic modulus of starch gel decreased with the increase in TP concentration during the same storage period. The ΔH_r_ of starch decreased and the aging rate decreased significantly. The gel texture became softer and the hardness value decreased, indicating that the addition of TP hindered the aggregation and rearrangement of amylose and reduced the intermolecular force of starch, thus weakening the degree of starch aging. The results of FTIR showed that the values of R1054/1021 and R995/1022 decreased gradually, which proved that TP could inhibit the cross-linking between starch molecules and reduce the relative order degree of starch. XRD results show that TP reduces the crystallinity of starch by reducing the rearrangement and crystallization of WS. The content of bound water and unmovable water increases, while the content of free water decreases. The addition of TP significantly improved the structure of the gel and inhibited the retrogradation of the gel. TP can delay the short- and long-term aging of WS to a certain extent. This not only realizes the development and utilization of TP value, but also provides a theoretical basis for the storage of starch-based foods.

## Figures and Tables

**Table 1:** Thermodynamic characteristics of gelatinization and retrogradation of WS with different concentrations of TP.

TP (% *w*/*w*)	Storage Time/d	T_0_/°C	T_p_/°C	ΔH/J·g^−1^	Retrogradation Percentage/%
0%	0	56.01 ± 0.11 ^c^	61.50 ± 0.36 ^a^	2.89 ± 0.02 ^a^	
	7	43.35 ± 0.07 ^h^	54.27 ± 0.32 ^cd^	1.59 ± 0.01 ^f^	55.00 ± 0.08 ^c^
	14	42.39 ± 0.14 ^i^	53.59 ± 0.20 ^d^	1.99 ± 0.07 ^e^	68.87 ± 0.10 ^a^
0.5%	0	56.07 ± 0.21 ^c^	61.60 ± 0.15 ^a^	2.38 ± 0.01 ^b^	
	7	43.58 ± 0.09 ^gh^	54.58 ± 0.40 ^bc^	1.06 ± 0.01 ^ij^	44.54 ± 0.03 ^g^
	14	43.40 ± 0.24 ^h^	54.32 ± 0.49 ^cd^	1.33 ± 0.02 ^g^	55.85 ± 0.12 ^b^
1%	0	56.33 ± 0.08 ^bc^	61.63 ± 0.06 ^a^	2.26 ± 0.03 ^c^	
	7	43.88 ± 0.12 ^fg^	54.64 ± 0.44 ^bc^	0.98 ± 0.01 ^k^	43.36 ± 0.10 ^h^
	14	43.67 ± 0.29 ^fgh^	54.47 ± 0.61 ^bc^	1.23 ± 0.01 ^h^	54.43 ± 0.05 ^d^
2%	0	56.50 ± 0.03 ^b^	61.69 ± 0.19 ^a^	2.15 ± 0.01 ^d^	
	7	44.36 ± 0.24 ^d^	54.86 ± 0.13 ^bc^	0.83 ± 0.02 ^l^	38.20 ± 0.06 ^i^
	14	43.97 ± 0.16 ^ef^	54.56 ± 0.36 ^bc^	1.10 ± 0.02 ^i^	51.17 ± 0.19 ^e^
3%	0	57.46 ± 0.18 ^a^	61.92 ± 0.36 ^a^	2.03 ± 0.01 ^e^	
	7	44.62 ± 0.16 ^d^	55.15 ± 0.17 ^b^	0.76 ± 0.03 ^l^	37.44 ± 0.06 ^i^
	14	44.29 ± 0.33 ^de^	54.94 ± 0.65 ^bc^	1.02 ± 0.05 ^jk^	50.25 ± 0.41 ^f^

Data are presented as mean ± standard deviation, *n* = 3. Data in the same column with different lowercase superscripts are significantly different (*p* < 0.05).

**Table 2:** Short-range order of retrogradation WS/different concentrations of the TP mixed system.

TP (% *w*/*w*)	Storage Time/d	R (1054/1021) cm^−1^	R (995/1022) cm^−1^
0	1	1.116 ± 0.01 ^def^	1.323 ± 0.01 ^efg^
	7	1.142 ± 0.01 ^d^	1.344 ± 0.02 ^de^
	14	1.214 ± 0.01 ^ab^	1.424 ± 0.01 ^a^
0.5	1	1.107 ± 0.01 ^efg^	1.315 ± 0.02 ^efg^
	7	1.135 ± 0.01 ^d^	1.332 ± 0.01 ^ef^
	14	1.236 ± 0.02 ^a^	1.415 ± 0.02 ^a^
1	1	1.097 ± 0.01 ^fgh^	1.306 ± 0.03 ^fg^
	7	1.126 ± 0.01 ^de^	1.327 ± 0.01 ^efg^
	14	1.219 ± 0.02 ^ab^	1.401 ± 0.01 ^ab^
2	1	1.082 ± 0.01 ^gh^	1.303 ± 0.02 ^fg^
	7	1.098 ± 0.01 ^fgh^	1.314 ± 0.02 ^fg^
	14	1.200 ± 0.01 ^bc^	1.384 ± 0.01 ^g^
3	1	1.076 ± 0.01 ^h^	1.298 ± 0.01 ^d^
	7	1.083 ± 0.02 ^gh^	1.303 ± 0.00 ^fg^
	14	1.186 ± 0.02 ^c^	1.370 ± 0.01 ^cd^

All data represent the mean of triplicates. Means within a column with different letters are significantly different (*p* < 0.05).

**Table 3:** T_2_ values of retrogradation WS different concentrations of TP mixed systems.

TP (% *w*/*w*)	Storage Time/d	T_2b_	T_21_	T_22_	T_23_
0%	1	0.19 ± 0.01 ^b^	1.95 ± 0.20 ^a^	12.33 ± 1.08 ^a^	231.01 ± 0.00 ^a^
	7	0.22 ± 0.00 ^a^	1.75 ± 0.13 ^ab^	12.33 ± 1.08 ^a^	215.22 ± 7.23 ^b^
	14	—	1.75 ± 0.13 ^ab^	10.72 ± 1.00 ^ab^	200.92 ± 3.15 ^c^
0.5%	1	0.16 ± 0.02 ^c^	1.95 ± 0.20 ^a^	10.72 ± 1.00 ^ab^	231.01 ± 0.00 ^a^
	7	0.22 ± 0.00 ^a^	1.52 ± 0.11 ^bc^	9.33 ± 0.90 ^b^	200.92 ± 3.15 ^c^
	14	—	1.52 ± 0.11 ^bc^	9.33 ± 0.90 ^b^	174.75 ± 0.00 ^d^
1%	1	0.16 ± 0.02 ^c^	1.75 ± 0.13 ^ab^	10.72 ± 1.00 ^ab^	215.22 ± 7.23 ^b^
	7	0.22 ± 0.00 ^a^	1.32 ± 0.00 ^cd^	9.33 ± 0.90 ^b^	200.92 ± 3.15 ^c^
	14	—	1.52 ± 0.11 ^bc^	9.33 ± 0.90 ^b^	174.75 ± 0.00 ^d^
2%	1	0.16 ± 0.00 ^c^	1.75 ± 0.13 ^a^	10.72 ± 1.00 ^ab^	200.92 ± 3.15 ^c^
	7	0.19 ± 0.01 ^b^	1.15 ± 0.00 ^d^	9.33 ± 0.90 ^b^	174.75 ± 0.00 ^d^
	14	—	1.32 ± 0.00 ^cd^	9.33 ± 0.90 ^b^	174.75 ± 0.00 ^d^
3%	1	0.16 ± 0.00 ^c^	1.75 ± 0.13 ^ab^	10.72 ± 1.00 ^ab^	200.92 ± 3.15 ^c^
	7	0.19 ± 0.01 ^b^	1.15 ± 0.00 ^d^	9.33 ± 0.90 ^b^	174.75 ± 0.00 ^d^
	14	—	1.15 ± 0.00 ^d^	9.33 ± 0.90 ^b^	174.75 ± 0.00 ^d^

Data are presented as mean ± standard deviation, *n* = 3. Values followed by different superscripts in the same column indicate significant differences (*p* < 0.05).

## Data Availability

The data used to support the findings of this study can be made available by the corresponding author upon request.
